# Low Power CMOS-Based Hall Sensor with Simple Structure Using Double-Sampling Delta-Sigma ADC

**DOI:** 10.3390/s20185285

**Published:** 2020-09-16

**Authors:** Ju Yong Lee, Younggyun Oh, Sein Oh, Hyungil Chae

**Affiliations:** 1 Department of Electrical and Electronic Engineering, Konkuk University, Seoul 05029, Korea; yoeung131@konkuk.ac.kr; 2 Department of Electrical and Electronic Engineering, Kookmin University, Seoul 02707, Korea; ohyounggyun@kookmin.ac.kr (Y.O.); chinig@kookmin.ac.kr (S.O.)

**Keywords:** hall sensor, double sampling, delta-sigma ADC, horizontal hall device, magnetic sensor

## Abstract

A CMOS (Complementary metal-oxide-semiconductor) Hall sensor with low power consumption and simple structure is introduced. The tiny magnetic signal from Hall device could be detected by a high-resolution delta-sigma ADC in presence of offset and flickering noise. Also, the offset as well as the flickering noise are effectively suppressed by the current spinning technique combined with double sampling switches of the ADC. The double sampling scheme of the ADC reduces the operating frequency and helps to reduce the power consumption. The prototype Hall sensor is fabricated in a 0.18-µm CMOS process, and the measurement shows detection range of ±150 mT and sensitivity of 110 µV/mT. The size of active area is 0.7 mm^2^, and the total power consumption is 4.9 mW. The proposed system is advantageous not only for low power consumption, but also for small sensor size due to its simplicity.

## 1. Introduction

A Hall sensor is used in various applications such as magnetic field measurement, automotive industry, consumer electronic products, etc. [1,2]. When a magnetic field is applied in the direction perpendicular to the moving direction of electric charges flowing in a conductor or semiconductor, Lorentz force is generated in the direction perpendicular to the direction of the electric charge movement. This phenomenon is known as Hall effect and the resulting output signal in type of either voltage or current is called Hall signal. A Hall sensor is a kind of magnetic sensor utilizing the aforementioned Hall signal, and the key component of a Hall sensor that converts an external magnetic field into voltage or current output signal is a Hall device. A Hall sensor consisting of a Hall device and other read-out circuits can be fabricated on semiconductor material including bipolar or CMOS (Complementary metal-oxide-semiconductor) processes. In particular, a CMOS-based Hall sensor is widely used due to the advantages of small size, low cost, and high reliability, etc. [3,4]. However, the Hall device in a CMOS process often suffers from offset and sensitivity problems. Large offset occurs due to process non-idealities, such as local difference in doping concentration, hole electrode asymmetry, package stress, and other process-voltage-temperature (PVT) variations, and it interferes with the detection of small input signals. Another shortcoming of a CMOS Hall device is low sensitivity which is mainly affected by the material characteristic and it leads to weak output signal. Commercial CMOS processes do not give designers the freedom to choose materials, so instead, sensitivity is generally enhanced by modifying the Hall device shape. Various Hall device structures such as four contact (4F) or multiple-terminal ones [5,6,7] have been attempted, but the improvement is not significant due to limitation on the material used. Therefore, the amplification process in a Hall sensor is essential to achieve a high signal-to-noise ratio (SNR), and a low-noise operational amplifier (op-amp) is very often used in the system. However, an op-amp in a CMOS process suffers from the trade-off between noise and power consumption, and the performance of an op-amp is vulnerable to PVT variation. The bigger difficulty with amplification is that the gain can be limited due to the large offset. This must be handled using a filter at expense of additional power consumption and hardware size.

**Figure 1:**
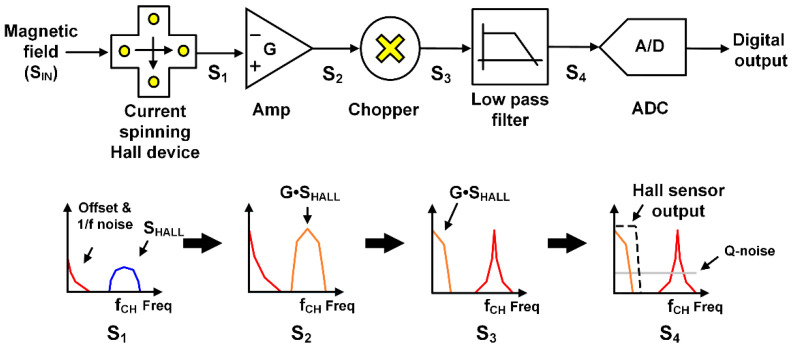
Conventional Hall sensor system configuration and signal processing in frequency domain.

A conventional Hall sensor system that overcomes sensitivity and offset problems is shown in Figure 1. An external magnetic field generates a Hall signal (S_HALL_), and the current spinning technique [6,8] is applied to a Hall device to separate S_HALL_ from offset and flickering (*1/f*) noise (from the Hall device) on the frequency domain, e.g., offset and *1/f* noise at DC and S_HALL_ at chopping frequency *f_CH_*. S_HALL_ usually goes to a higher frequency region to minimize the effect of *1/f* noise arising from the following blocks. Then, S_HALL_ as well as offset and *1/f* noise are amplified by a gain of G and their positions in the frequency domain are switched by a chopper. Finally, the undesired components at *f_CH_* are attenuated by a low pass filter and the following analog-digital converter digitalizes only the input related signal. Although this conventional configuration is effective for achieving high SNR by suppressing offset and *1/f* noise, its circuit implementation is very complex and the hardware size as well as power consumption increase significantly. Furthermore, the addition of other high-resolution techniques such as auto-zero technique, correlated double sampling, or switched biasing amplifier will make things worse [9,10,11,12].

**Figure 2:**
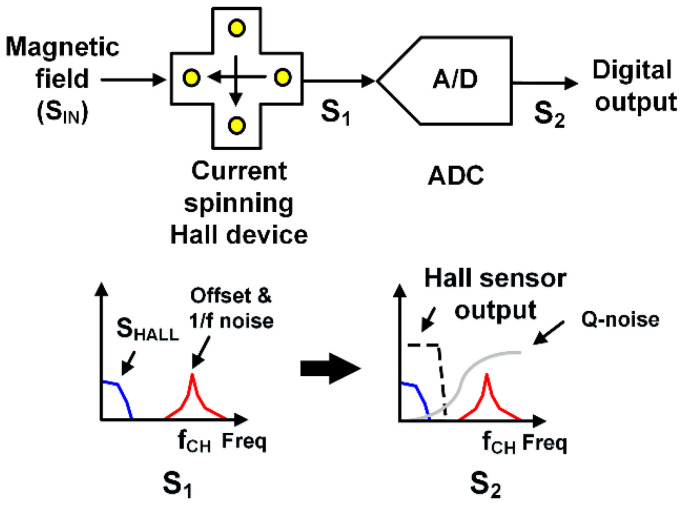
Proposed Hall sensor system configuration using Hall device and high-resolution delta-sigma ADC.

In this work, we propose a new Hall sensor system architecture consisting of only a Hall device and a high-resolution double sampling discrete-time delta-sigma ADC. The Hall sensor system can be extremely simplified by handling the small Hall signal (S_HALL_) directly with a high-resolution ADC with inherit chopper stabilization. As in Figure 2, S_HALL_ is located around DC while the offset and *1/f* noise are translated to higher frequency by choosing a different current spinning direction to that shown in Figure 1. Then, the weak S_HALL_ can be detected by a noise-shaping ADC regardless of the existence of the offset and *1/f* noise, which can significantly reduce circuit complexity, area, and power consumption.

## 2. Proposed Hall Sensor Architecture

### 2.1. Hall Device Structure and Current Spinning

**Figure 3:**
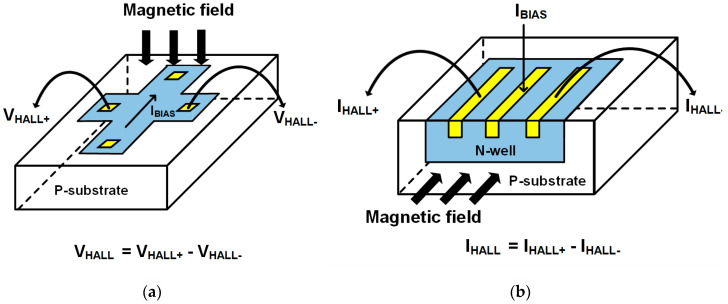
(**a**) Horizontal-type Hall device (**b**) Vertical-type Hall device.

There are two types of Hall devices depending on the signal direction: a horizontal type Hall device (HHD) and a vertical type Hall device (VHD), and Figure 3 shows popular structures of an HHD and a VHD. In case of an HHD in Figure 3a, the magnetic field is applied in the vertical direction to P-substrate and the Hall signal shows as voltage output V_HALL_, which is called Hall voltage and formed in the vertical direction to the magnetic field. For a VHD, the magnetic field is applied in the horizontal direction to P-substrate as in Figure 3b, and the Hall signal is formed in current type (I_HALL_). Due to the short circuit effect, the sensitivity of VHDs is usually lower than that of HHDs [5,7], so an HHD is used in this work to achieve high sensitivity. Many HHD structures are introduced so far, and among them, we use a cross-sectional structure since it is the most optimized one for the sensitivity [13,14].

**Figure 4:**
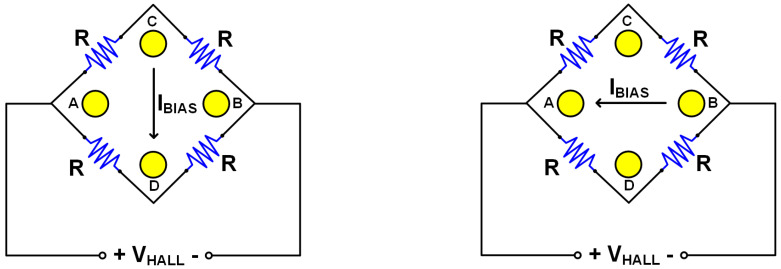
Modeling of ideal Hall device with wheastone bridge circuit.

**Figure 5:**
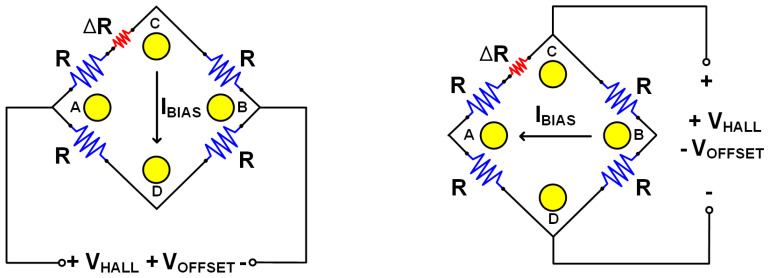
Modeling of practical Hall device and current spinning technique applied to it.

However, the cross-sectional HHD suffers from an offset problem like other kinds of Hall devices due to process non-idealities. There are several circuit techniques removing offset of a Hall device, and the current spinning technique is the most widely used one due to its high efficiency. The current spinning technique models a Hall device with a Wheatstone bridge circuit consisting of four resistors (R). When the Hall device is ideal, all resistors have the same resistance and no offset occurs. Also, due to the perfect symmetry, the Hall voltage (V_HALL_) would be the same regardless of the current direction as in Figure 4. If there is any asymmetry in a Hall device caused by non-idealities, the four-resistor model will have different resistances and it can be modeled with a small resistance (∆R) as in Figure 5. This difference generates offset voltage at the output, but it can be cancelled out by switching the current direction. The offset voltage alternates its polarity depending on the current direction as in Figure 5 while V_HALL_ remains the same as mentioned above. Therefore, periodic change of the current direction modulates only the offset voltage(+/−V_OFFSET_) and translates it from DC to high frequency band. At the same time, the current spinning is also applied to *1/f* noise from the Hall device, which moves to high frequency band along with the offset. This allows for the isolation of unwanted signals. However, V_OFFSET_ is often larger than V_HALL_, so complicated signal processing may be necessary after the isolation.

### 2.2. Directly Connected Hall Device and High-Resolution Discrete-Time Delta-Sigma ADC

**Figure 6:**
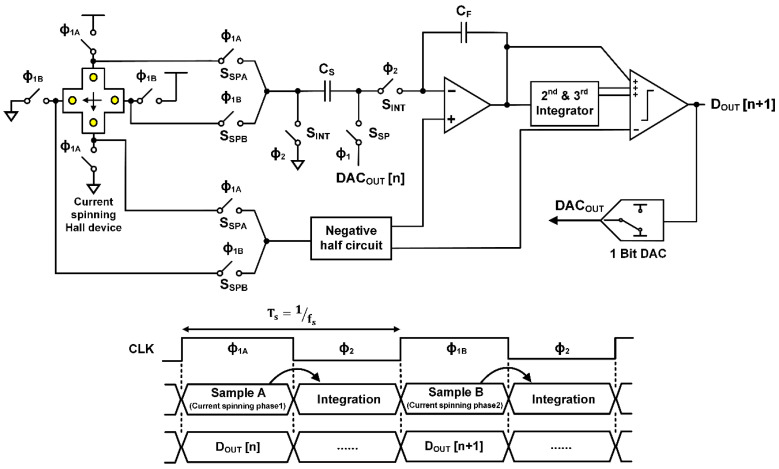
Direct connection of CMOS Hall device and high-resolution delta-sigma ADC, and operation timing diagram of Hall sensor.

A conventional Hall sensor requires many blocks to remove the offset and *1/f* noise, which increases power consumption, chip area, and design complexity. To mitigate the problem, we propose a simple Hall sensor architecture composed of only a Hall device and an ADC avoiding the use of many complicated signal processing blocks such as an amplifier and a filter. However, if a Nyquist rate ADC is used as in a conventional Hall sensor system, even though the offset and *1/f* noise are isolated by current spinning, an ADC with a very high-resolution is necessary to detect the small Hall voltage, which can make things worse since the power consumption increases four times faster than the resolution. A delta-sigma ADC can be a good option since it can digitize only the Hall voltage with high-resolution (more than 15-bits) with the help of oversampling and noise shaping [15,16,17]. A Hall device and a delta-sigma ADC can be easily combined keeping the current spinning capability. By spinning the bias current direction according to the sampling frequency of a discrete-time delta-sigma ADC, the offset and *1/f* noise are moved to the Nyquist frequency while the Hall voltage stays within the band of the ADC. The proposed system and operation timing diagram are shown in Figure 6. The ADC samples the Hall voltage on C_s_ during half of the sampling period (Phase ϕ_1_), and the sampled signal is processed by the first integrator for the next half of the period (Phase ϕ_2_). The first integrator output is passed to the next stages sequentially, and the final digital output (D_OUT_) is obtained at the comparator every sampling clock period. For current spinning, the Hall device changes its connection (ϕ_1A,_ ϕ_1B_) every sampling clock period as in Figure 6, and the sampling switches (S_SPA_, S_SPB_) at the ADC input are turned on and off accordingly. Then, the sampled input signal is integrated by common integration switches (S_INT_). This configuration is advantageous to achieve high SNR as long as the oversampling ratio is high enough to prevent noise folding problem. However, for a given signal bandwidth and SNR, the increase of oversampling ratio will increase the power consumption of a delta-sigma ADC, and the total power saving might not be substantial compared to a conventional Hall sensor system.

### 2.3. Double Sampling Delta-Sigma ADC

**Figure 7:**
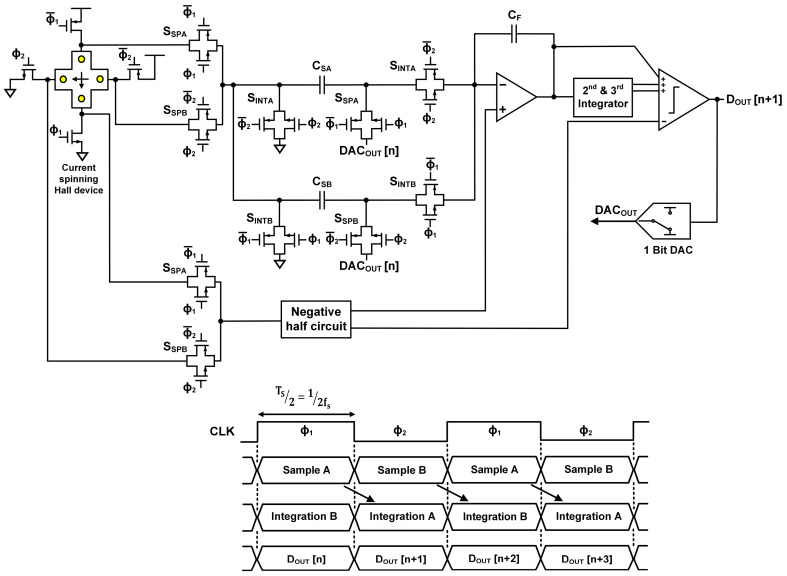
Hall sensor using double sampling delta-sigma ADC, and its operation timing diagram.

In the proposed Hall sensor system above, the power consumption is now dominated by the high-resolution delta-sigma ADC with a high oversampling ratio. For a given signal bandwidth, increasing the oversampling ratio requires a very high sampling frequency, resulting in high power consumption due to discrete-time operation. Therefore, the ADC operation speed should be optimized to achieve good power efficiency of the whole system, and a very effective but simple solution is using double sampling scheme [18]. In a double sampling system, the input is sampled at every phase of the sampling clock, and the sampling frequency becomes doubled compared to that with conventional sampling. In other words, the sampling clock speed could be halved for a given signal bandwidth, and the power consumption can be reduced substantially as well. Speed of a discrete-time delta-sigma ADC is mostly limited by the settling time of an integrator that dominates the whole power consumption, and effectively doubled settling time of integrators is allowed in the double sampling scheme. Therefore, a double sampling delta-sigma ADC is often used in sensor applications where high-resolution and low power consumption are critical [19,20]. The proposed double sampling Hall sensor system and its operation timing diagram are shown in Figure 7. The ADC includes two sets of track-and-hold (T/H) circuits, and in Phase ϕ_1_, the Hall voltage is sampled on C_SA_ while the previously sampled signal on C_SB_ is integrated by the first integrator. In the next phase (Phase ϕ_2_), C_SB_ now samples the Hall voltage, and at the same time, the signal on C_SA_ gets integrated. This parallel sampling-integration process by the two T/H circuits is repeated, and the first integrator output is passed to the next integrator every half clock phase (*T_S_/2*). After the sequential operation, the final digital output (D_OUT_) is obtained at the comparator output which runs at the speed of 2*f_S_*. In addition to the advantage of speed increase (or power saving), the current spinning becomes even easier in the proposed system due to the two T/H circuits. The two sets of outputs of the Hall device only need to be each connected to the sampling switches (S_SPA_, S_SPB_), and no additional circuitry is necessary to isolate the offset and noise. Therefore, the proposed Hall sensor system architecture not only reduces the power consumption, but also substantially simplifies the hardware implementation.

## 3. Circuit Implementation

### 3.1. Cross-Sectional Horizontal Hall Device

**Figure 8:**
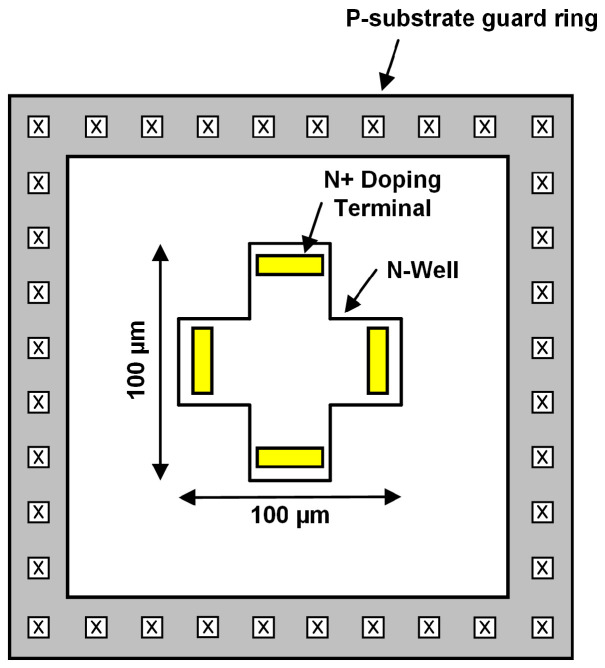
Cross-sectional Horizontal-type CMOS Hall device layout.

A cross-sectional HHD is chosen as mentioned in Section 2.1 due to its relatively high sensitivity compared to other structures, and it is designed in a CMOS process. Although the HHD’s offset can be eliminated by current spinning technique, it still exists at the HHD’s output and limits the input range of a high-resolution delta-sigma ADC. Therefore, the layout is carefully optimized to minimize the offset and maximize the sensor dynamic range. A perfectly symmetrical cross-shaped Hall device is surrounded by a wide guard ring to minimize external influences on the offset as shown in Figure 8. The Hall device itself is made of N-well layer on P-substrate and has a size of 100 × 100 µm optimized for offset [13,14]. There are 4 terminals based on N+ doping for current spinning of the Hall device, and the length of metal wires connected to the terminals as well as switches are also balanced.

### 3.2. 3rd-Order Discrete-Time Delta-Sigma ADC

**Figure 9:**
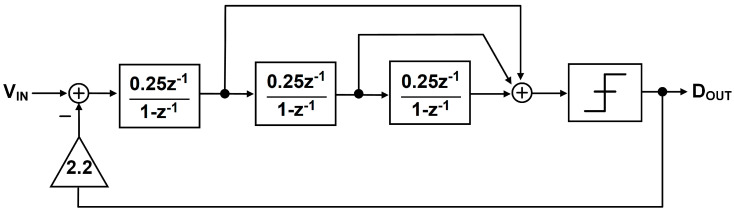
3rd-order Discrete-time Delta-Sigma ADC block diagram.

A 3rd-order discrete-time delta-sigma ADC is used in the proposed system to achieve more than 15-bit resolution, and the structure is shown in Figure 7 and Figure 9. It has CIFF structure for low power consumption and high linearity [21]. There are three integrators for 3rd-order noise shaping and the quantizer performs 1bit quantization. The integrator and DAC coefficients are optimized for high dynamic range and for easy signal summation before the quantization, and the resulting noise transfer function of the ADC is as Equation (1).

(1) $$\mathrm{NTF}\;=\;\frac{{{(1\;-\;z}^{-1})}^{3}}{1-\frac{49}{20}z^{-1}\;+\;\frac{163}{80}z^{-2}\;-\;\frac{177}{320}z^{-3}}$$

The three poles are located at (0.5822 + *j*0), (0.934 − *j*0.2792), (0.934 + *j*0.2792) in pole-zero plot, and they are all inside the unit circle and the stability is ensured. The simulation shows an SNDR of 92 dB when the oversampling ratio is 128.

**Figure 10:**
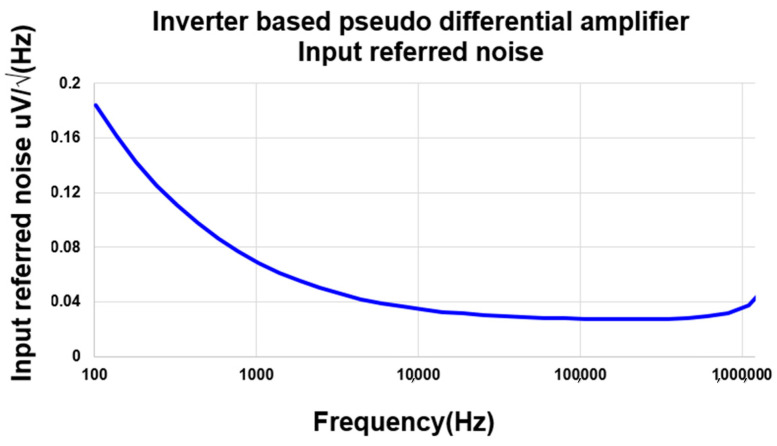
Input referred noise vs. frequency plot of amplifier.

The integrators are based on switched-capacitor amplifiers, and the circuit implementation of the amplifier is shown in Figure 10. Requirement for the amplifier is very high due to the target resolution, and a pseudo differential amplifier consisting of inverters is used instead of a conventional operational amplifier which is often energy inefficient [22,23,24,25]. The pseudo differential amplifier has a very simple structure and has two complementary source-coupled pairs for low power consumption. The gain of the amplifier can be derived as Equation (2).

Gain = (g_mn1,2_ + g_mp1,2_)(r_on1,2_//r_op1,2_) (2)

where g_mn1,2_ and g_mp1,2_ represent the transconductance of the complementary input devices (M_N1,2_ and M_P1,2_) respectively, and r_on1,2_ and r_op1,2_ represent the output resistance of them. The input devices have length of >10 µm to achieve DC gain over 60 dB in a typical condition that is enough for the ADC target performance. Figure 10 shows the input referred noise curve of the amplifier, and the integrated input referred noise from 100 Hz to 7.81 KHz is simulated to be 5.24 µV_rms_, which corresponds to 0.508 µT in our system. Therefore, noise from the amplifier is low enough not to affect the overall system resolution, and the thermal noise from the Hall device becomes the main noise source.

The common mode feedback circuit consists of switched capacitors as conventional ones [26]. Due to double sampling scheme, there are two sets of feedback circuits since the common mode feedback requires two phases of operation. One set is initialized to VCM_CMFB_ during sampling while the other set detects the common mode output level of the amplifier and performs feedback operation during integration.

**Figure 11:**
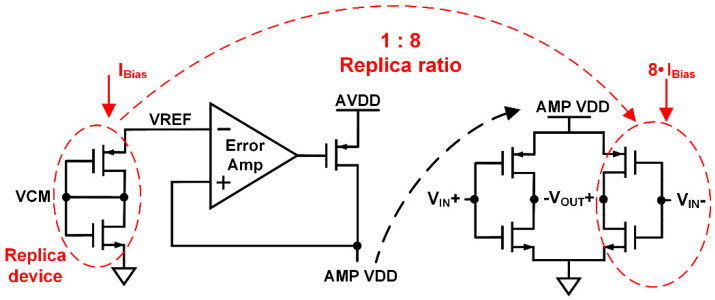
Adaptive LDO for amplifier supply voltage and replica circuit for reference voltage.

**Figure 12:**
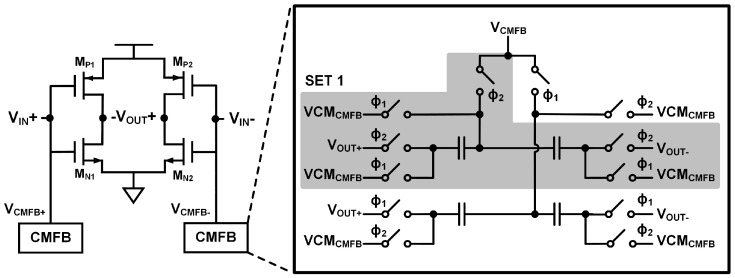
Inverter based pseudo differential amplifier and switched-capacitor common mode feedback circuit.

This kind of amplifiers can be prone to PVT variations and the gain variation significantly affects the overall performance. Also, the pseudo differential structure can lead to overcurrent flowing due to absence of the tail current. Therefore, an adaptive biasing scheme is used for robustness to variations. An adaptive LDO shown in Figure 11 provides supply voltage (AMP VDD) to the amplifier, and the reference voltage is generated by applying a bias current to a diode-connected self-biasing replica circuit. The diode-connected replica devices are reduction of the input devices of the amplifier to 8:1 ratio for low static power consumption. The bias current comes from a constant-g_m_ block, so the adaptive LDO provides AMP VDD that guarantees the stable gain of the amplifier. When this biasing scheme is used for the amplifier, the common mode voltage (VCM) of the integrator needs to be same to the gate voltage of the replica devices. Therefore, the common mode reference voltage (VCM_CMFB_) in Figure 12 is also generated by the adaptive LDO.

The first integrator dominates the overall performance of a high-resolution delta-sigma ADC, so the design of a T/H circuit at the input of the first integrator is also important. A bootstrapping switch is used at the front end in conventional ADCs, however the output voltage of a Hall device is mostly tiny and the offset is also further reduced by a careful layout design [27]. Therefore, a transmission gate is used for the input switch instead of a bootstrapping one for simplicity without affecting linearity.

**Figure 13:**
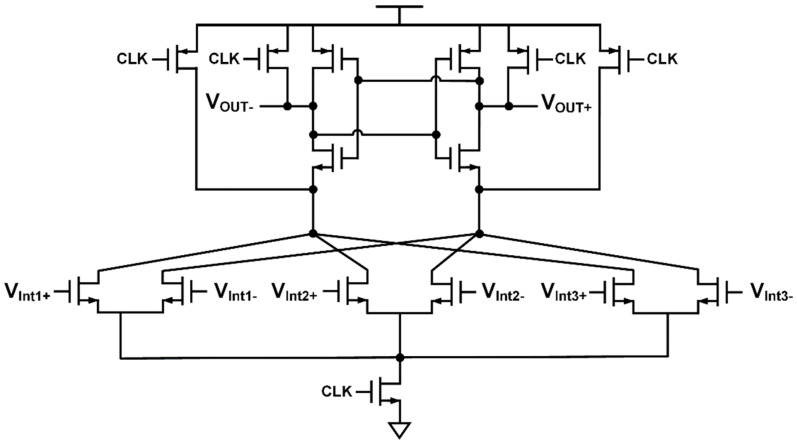
Multi-input comparator summing three integrator output signals.

After the third integrator, a single-bit quantizer is used instead of a multi-bit quantizer to avoid the complexity of the system since digital calibration is essential for multi-bit quantization for linearity. A multi-input comparator in Figure 13 not only performs the single-bit quantization, but also replaces a summing amplifier that combines three integrator outputs. The gain of each integrator is set to 1/4 as in Figure 9 in order to keep the summing factors of the three paths to unity. Therefore, the comparator input devices are sized exactly the same, and the mismatch effect can be minimized.

**Figure 14:**
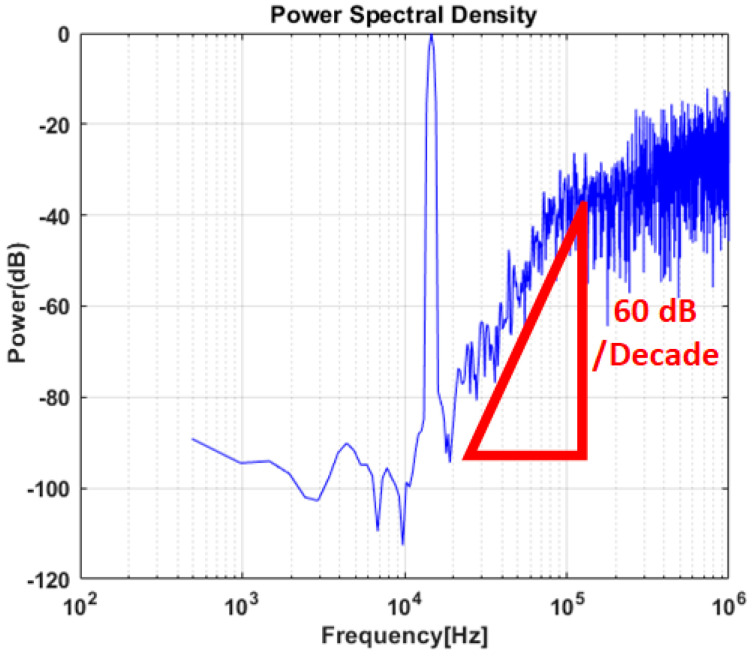
Simulated output power spectral density of standalone delta-sigma ADC.

The standalone ADC is designed in 0.18 µm CMOS process, and the simulated output power spectral density is shown in Figure 14. The simulated SNDR is 92 dB, and the NTF slope is 60 dB/decade as expected. The sampling frequency is 2 MHz, and the power consumption is 4.5 mW.

## 4. Results

**Figure 15:**
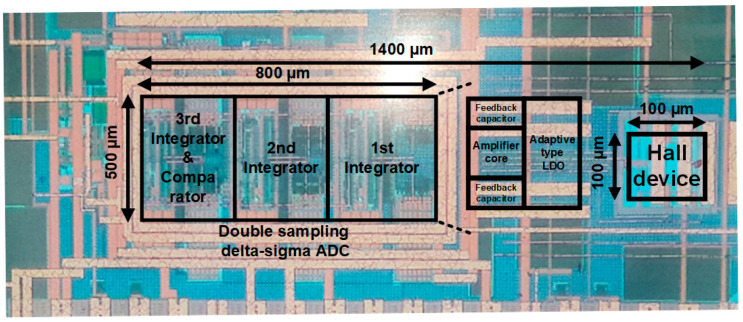
Die photograph of Hall sensor prototype fabricated in 0.18 µm CMOS process.

The proposed Hall sensor system was implemented and fabricated in 0.18 µm CMOS process. The die photograph of the prototype is shown in Figure 15 and the active area including the high-resolution double sampling discrete-time delta-sigma ADC and the horizontal Hall device is 1400 µm by 500 µm. Most of the area is occupied by the switched capacitors that are designed for high resolution.

**Figure 16:**
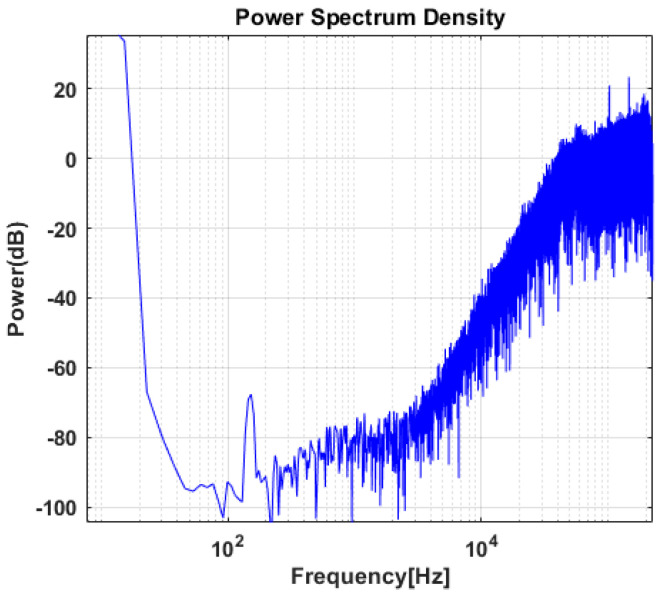
Measured output power spectral density of prototype when DC magnetic field of 150 mT is applied.

**Figure 17:**
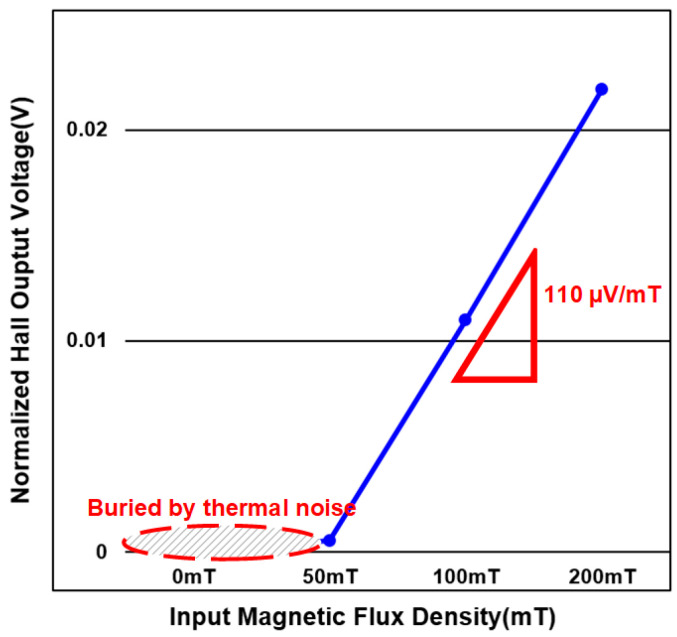
Normalized Hall output voltage versus input magnetic flux density from measurement of Hall sensor prototype.

An external magnetic field is generated and applied to the prototype in a vertical direction to the sensor surface, and the 1-bit digital output stream is obtained at the ADC output. Figure 16 shows the output power spectral density when DC magnetic field of 150 mT is applied. The 1-bit digital output signal is digitally processed so that the out-of-band noise is filtered out. The final output value representing the input magnetic flux density is shown in Figure 17.

When the magnetic flux density varies from zero to 200mT, it is observed that the output value is proportional to the input signal. The measured sensitivity of the whole system is 110 µV/mT, and the detectable input range that guarantees high linearity (>99%) is measured to be ±150 mT. Although the ADC input becomes saturated when the magnetic flux density is greater than 200 mT, the detection range of the prototype is enough since the magnetic field is often smaller than that in most applications. The total power consumed by the ADC and the Hall device is measured to be 4.9 mW. Table 1 summarized the performance of our prototype and shows comparison with other works [28,29]. Our proposed Hall sensor system consumes much less power than others without affecting the linearity. The detectable range is reduced by a small bit due to the direct connection of the Hall device and the ADC, but accurate measurement is possible with a minimized hardware not using any complicated calibration technique.

In conclusion, a CMOS Hall sensor system consisting of only a Hall device and a high-resolution delta-sigma ADC is proposed. The double sampling scheme for the ADC combined with current spinning technique substantially suppresses the offset from the Hall device and also reduces the power consumption by halving the required clock frequency of the ADC. The proposed Hall sensor structure is helpful for low power consumption and can have much smaller hardware size due to its simplicity. The measurement of the prototype Hall sensor shows better power efficiency and smaller size compared to existing works, and the proposed technique can be useful in many IoT applications that require magnetic field measurement.

## Figures and Tables

**Table 1:** Performance summary and comparison with other CMOS Hall sensors.

Parameter	[28]	[29]	This Work
Technology	CMOS 0.5 µm	CMOS 0.8 µm	CMOS 0.18 µm
Supply Voltage	5 V	5 V	2.2 V
Power Consumption	21 mW	20 mW	4.9 mW
Measurement Range	±10.8 mT	±175 mT	±150 mT
Offset	3.65 µT	0.48 mT	16 µT
Linearity	N/A	>99%	>99%
Area	2.9 mm^2^	1 mm^2^	0.7 mm^2^
